# Risks and Protective Factors Associated to Homophobic Cyberbullying Among Youth

**DOI:** 10.1002/ab.70034

**Published:** 2025-05-21

**Authors:** Alberto Amadori, S. Henry Sherwood, Stephen T. Russell, Antonella Brighi

**Affiliations:** ^1^ Faculty of Education Free University of Bozen‐Bolzano Bolzano Italy; ^2^ Department of Human Development and Family Sciences University of Texas at Austin Austin Texas USA

**Keywords:** adolescence, homophobic cyberbullying, peer norms, social dominance orientation, socio‐emotional competencies

## Abstract

Homophobic cyberbullying and other forms of anti‐LGBTQ+ bias among adolescents are an emerging and concerning form of online bias‐based aggression. However, little research has explored its prevalence and correlates. This study aims to address this gap by investigating homophobic cyberbullying through the theoretical lens of a socioecological stigma framework. Specifically, it examines the association between individual factors (socio‐emotional competencies), contextual factors (homophobic social norms), and homophobic cyberbullying. Additionally, it explores the moderating effect of socio‐emotional competencies on the relationship between homophobic social norms and social dominance orientation on homophobic cyberbullying. Parallel (in‐school and online) survey samples (*N* = 3807) were collected among Italian youth (*M*_age_ = 16.69; SD = 1.97). A series of multiple linear regression models with two‐way and three‐way interaction effects were tested. Descriptive results indicated that heterosexual boys reported higher rates of homophobic cyberbullying. The regression analysis demonstrated that socio‐emotional competencies were negatively associated with homophobic cyberbullying, whereas homophobic social norms were positively related to it. Furthermore, socio‐emotional competencies mitigated the impact of homophobic social norms on the relationship between social dominance orientation and homophobic cyberbullying. The study underscores the urgent need for evidence‐based interventions that challenge and reshape gendered and heteronormative beliefs perpetuating homophobic cyberbullying, particularly among adolescent heterosexual boys, by encouraging critical reflection on masculinity and sexuality within educational settings and peer networks.

## 1 Risk and Protective Factors Associated With Homophobic Cyberbullying Among Youth

In recent years, scholars have increasingly examined the role of bias in cyberbullying, including how social identities shape online aggression (Strohmeier et al. 2022; Zych and Llorent 2023). However, while general research on cyber‐hate—which encompasses online harassment based on race, religion, gender, and sexual orientation (Wachs et al. 2019)—has grown, less attention has been paid to homophobic cyberbullying as a distinct form of bias‐driven aggression. Homophobic cyberbullying is not just a subset of cyber‐hate; it is a unique manifestation of anti‐LGBTQ+ bias that operates at both individual and structural levels. Specifically, it intersects with broader societal prejudices against LGBTQ+ individuals, targeting them based on real or perceived sexual orientation, gender identity, or expression. Wachs et al. (2019) extensive work on predictors of cyber‐hate emphasizes that hate‐based online behaviors are driven by a combination of individual traits and broader social influences, which similarly apply to homophobic cyberbullying. However, while cyber‐hate can target various aspects of identity, homophobic cyberbullying specifically involves attacks on individuals' real or perceived sexual orientation, which makes it particularly salient in discussions of prejudice against LGBTQ+ youth.

Homophobic cyberbullying involves targeted attacks stemming from societal prejudices against sexual minorities, amplifying traditional power imbalances through online platforms. Acts of homophobic cyberbullying—such as spreading derogatory rumors or threatening messages related to sexual orientation—can have devastating effects on LGBTQ+ youth, who already face heightened risks of marginalization in both online and offline environments (Abreu and Kenny 2017; Elipe et al. 2018). In fact, national statistics reveal that LGBTQ+ youth are disproportionately affected by cyberbullying. A report by the Human Rights Campaign and Human Rights Campaign Foundation (2020) in the United States found that LGBTQ+ youth are almost twice as likely to experience cyberbullying as their heterosexual peers. Additionally, a systematic review showed that the prevalence of cyberbullying among LGBTQ+ youth varies significantly, ranging from 10.5% to 71.3% (Abreu and Kenny 2017). Research conducted among Spanish adolescents further underscores this disparity, revealing that 41.1% of sexual minorities reported online victimization due to their sexual orientation, compared to just 3% of their heterosexual peers (Gámez‐Guadix and Incera 2021).

This study examines the mechanisms driving homophobic cyberbullying, focusing on homophobic social norms (HSN), social dominance orientation (SDO), and the buffering effect of socio‐emotional competencies (SEC) as protective factors. Understanding the factors that contribute to this form of online aggression is crucial, not only for advancing theoretical knowledge but also for developing effective strategies to reduce its impact. Schools, policymakers, and digital platforms need clearer guidance on how to address homophobic cyberbullying and create safer online environments.

### 1.1 The Moderating Effect of Socio‐Emotional Competencies on Aggressive Behavior

The moderating role of SEC in aggressive behavior during adolescence has been widely explored, emphasizing its importance in managing conflict and fostering positive social interactions (Malti and Song 2018). SEC includes skills such as emotional regulation, empathy, and social awareness, which are crucial in managing impulses and fostering positive social interactions. Adolescents with higher levels of SEC are generally better equipped to handle interpersonal tensions without resorting to aggression, including cyberbullying (Zych et al. 2018, 2019). For example, emotional regulation helps individuals manage their emotional responses, reducing the likelihood of reacting aggressively online. Studies have consistently shown that adolescents with poor emotional regulation are more prone to cyberbullying due to difficulties in controlling negative emotions like anger and frustration (Estévez et al. 2019; Gómez‐Ortiz et al. 2017). Similarly, empathy, another key component of SEC, has been found to reduce the likelihood of engaging in cyberbullying, as empathetic adolescents are more aware of the emotional impact their actions have on others (Brewer and Kerslake 2015). On the other hand, a lack of empathy has been linked to increased cyberbullying behaviors (Ang and Goh 2010). Furthermore, social awareness helps adolescents navigate social interactions more effectively, reducing the chances of conflict escalation and fostering a more inclusive social environment (Wang 2022). For instance, one study found that SEC moderated the impact of negative emotions on controlling behavior in adolescents (Rodríguez‐deArriba et al. 2023). Another investigation reported that emotional intelligence moderated the relationship between problematic internet use and cyberbullying perpetration, particularly among boys with lower levels of emotional intelligence (Yudes et al. 2022). Additionally, research has shown that emotional intelligence also moderated the persistence of dating violence perpetration over time, suggesting a buffering effect in preventing sustained aggression (Fernández‐González et al. 2018). Furthermore, other studies have indicated that high levels of empathy reduce the likelihood of adolescents engaging in homophobic cyberbullying, as empathetic individuals are more attuned to the emotional consequences of their actions (Wright and Wachs 2021).

While SEC has been shown to reduce general cyberbullying, its specific role in moderating bias‐based aggression, particularly homophobic cyberbullying, remains underexplored. This gap is critical because bias‐based cyberbullying is often driven by deeply entrenched social prejudices, meaning SEC may be less effective in challenging these behaviors than in reducing general aggression. Recent studies suggest that SEC interventions can reduce discriminatory behaviors (Wachs et al. 2023), but they may be less effective in altering deeply internalized biases. Given this, our study examines whether SEC moderates the relationship between HSN, SDO, and homophobic cyberbullying. If SEC buffers these effects, it would provide strong evidence for the importance of socio‐emotional learning programs in addressing bias‐driven cyberbullying (Amadori et al. 2023).

### 1.2 Peer Norms and Homophobia

When examining the role of social influence in aggressive behavior, existing research on cyberbullying has predominantly focused on the impact of peer norms (Lazuras et al. 2019; Nickerson et al. 2022). According to the Theory of Social Norms (Berkowitz 1972), the behaviors of youth are strongly influenced by peers' beliefs and actions. Higher levels of cyberbullying tend to coincide with greater perceived peer‐norm support for engaging in cyberbullying behaviors (Piccoli et al. 2020). Young people who engage in specific risky online behaviors, such as sharing personal information with strangers or cyberbullying, often perceive these actions as normative, believing that their peers not only approve but also participate in similar activities (Sasson and Mesch 2014).

Within the specific context of homophobia, studies suggest that adolescents' attitudes are influenced and subsequently modified by the homophobic attitudes displayed by their peers (la Roi et al. 2020; Poteat 2007), potentially superseding their own beliefs. For example, an investigation among male team sports participants found that prescriptive and proscriptive homophobic norms significantly predicted homophobic language use, outweighing individuals' beliefs (Denison et al. 2021). Thus, for adolescents, peer norms may play a key role in the perpetration of cyberbullying and homophobic cyberbullying.

### 1.3 Social Dominance Theory and Bias‐Based Cyberbullying

From a stigma‐based socioecological perspective (Earnshaw et al. 2018; Newman and Fantus 2015), an individual's beliefs about social hierarchy and inequality are shaped by both personal and contextual factors. A key framework contributing to this understanding is Social Dominance Theory, a psychological and sociopolitical model that explains how group‐based social hierarchies are maintained (Pratto et al. 1994). SDO reflects an individual's preference for group‐based dominance and inequality, with empirical evidence consistently supporting its role (Pratto et al. 2013; Sidanius and Pratto 1999). Prior research has shown that individuals with high levels of SDO are more prone to engaging in aggressive behaviors, including cyberbullying, especially when bias‐based factors are at play (Castellanos et al. 2023; Kuldas et al. 2022; Wang 2022).

SDO correlates with greater prejudice against the LGBTQ+ community, particularly among heterosexual men, who exhibit high levels of bias toward gay individuals (Licciardello et al. 2014; MacInnis and Hodson 2015; Puckett et al. 2017). In contexts with strong negative social norms toward homosexuality, individuals with high SDO may be more likely to engage in homophobic cyberbullying, as these norms reinforce their tendencies toward dominance and prejudice (O'Brien et al. 2013).

Past research found that social dominance goals predicted increased bullying in classrooms with stronger status hierarchies, while the influence of SDO on bullying diminishes in peer environments that do not encourage aggressive behaviors (Pan et al. 2023, 2020). This study focuses on how social norms interact with SDO to escalate homophobic behaviors in digital spaces, positing that elevated social norms around homophobia intensify the effect of SDO on such behaviors.

### 1.4 The Present Study

While research has documented the prevalence of bias‐based cyberbullying, a gap remains in understanding the factors contributing to online aggression targeting sexual orientation. We propose a conceptual model integrating SDO and SEC to examine how SEC moderates the relationship between stigma‐based socioecological factors, HSN, and SDO in homophobic cyberbullying. Existing literature has demonstrated that SDO is a key predictor of bias‐based aggression, as individuals with high SDO are more likely to engage in bullying that reinforces group‐based social hierarchies (Kuldas et al. 2022; Pratto et al. 1994). Similarly, HSN within peer groups contribute to the normalization and persistence of homophobic cyberbullying, reinforcing discriminatory behaviors online (Berkowitz 1972; Lazuras et al. 2019). However, little is known about protective factors that can mitigate these influences, particularly SEC. Prior research has shown that SEC can reduce aggressive behaviors, yet it remains unclear whether this buffering effect extends to bias‐driven cyberbullying, particularly in online spaces where discriminatory behaviors are more readily normalized. This study addresses this gap by testing whether SEC weakens the relationship between SDO, homophobic norms, and homophobic cyberbullying perpetration.

We propose a conceptual model integrating SDO and SEC to examine how SEC moderates the relationship between stigma‐based socioecological factors, HSN, and homophobic cyberbullying. Grounded in stigma‐based socioecological theory (Earnshaw et al. 2018), this study investigates how macro‐level biases, such as HSN, shape individual‐level attitudes and behaviors. Additionally, social norms theory (Berkowitz 1972) suggests that peer group attitudes significantly influence adolescents' engagement in cyberbullying, making it critical to examine how SEC might buffer these effects. Based on our conceptual model, we test the following hypotheses:

We expect that heterosexual boys, driven by peer norms and societal expectations, will report higher levels of homophobic cyberbullying compared to other groups.

We hypothesize that adolescents with stronger HSN will be more likely to engage in homophobic cyberbullying than those with weaker norms.

We anticipate that higher levels of SDO will be positively associated with increased homophobic cyberbullying, as individuals with greater social dominance tendencies are more likely to reinforce group‐based hierarchies through discriminatory online behaviors.

We hypothesize that SEC will moderate the relationship between SDO and homophobic cyberbullying, and that this moderation will vary depending on the level of HSN. Specifically, the effect of SDO on homophobic cyberbullying will be stronger in peer groups with high HSN, but this relationship will be weaker among adolescents with higher levels of SEC, as SEC buffers against negative peer influences.

## 2 Methods

### 2.1 Sample and Procedure

Data were collected from 3807 participants as part of the Online Wellbeing for Sexual and Gender Minority youth Project (OWSM), an explanatory mixed‐method research project on Italian adolescents (*M*_age_ = 16.69, SD = 1.97; girls = 50.6%, boys = 26.1%, other gender identities = 22.5%). Data collection involved 2 sampling methods: 2296 responses were obtained online, predominantly from LGBTQ+ youth, and an additional 1511 responses were gathered from 5 schools. Among LGBTQ+ youth, 32.1% identified as lesbian or gay, and 67.9% identified as bisexual, asexual, pansexual, queer, or questioning.

This study employed two distinct sampling methods. First, an anonymous survey was distributed online through social media platforms such as TikTok and Instagram. To reach a broad spectrum of youth, we sought the cooperation of nonprofit and LGBTQ+ organizations on social networks to assist in sharing the survey link. Given the sensitive nature of the LGBTQ+ topic, the study followed American Psychological Association guidelines that recommend waiving parental consent requirements for research involving LGBTQ+ youth (American Psychological Association 2018). In accordance with the European GDPR, individuals aged 14 and above were permitted to provide their informed consent to participate in the study. In the online sample (*M*_age_ = 17.09, SD = 2.08), 27.5% identified as boys, 39.6% as girls, and 32.9% as other gender identities (including nonbinary, trans, questioning). In terms of sexual orientation, 2.8% identified as heterosexual, while 83.2% identified as lesbian, gay, bisexual, pansexual, asexual, queer, or questioning.

Second, a separate survey was administered by inviting several high schools from northern Italy to participate in the OWSM project. In the five schools that agreed to participate, meetings were conducted to acquaint teachers with the study's purpose and the survey completion process. As the survey was conducted during school hours, parental consent was required for student participation. In the school sample (*M*_age_ = 16.09, SD = 1.61), 24% identified as boys, 67.5% as girls, and 4.6% as other gender identities. Most identified as heterosexual (52.5%), while 20.9% identified as a sexual minority. Missing data on gender and orientation ranged from 3.9% to 28.5%. Adolescents involved in the study completed the survey electronically. Importantly, the questionnaire's structure was consistent across both sampling methods, with a completion time of about 30 min. Both components of the study protocol adhered to ethical guidelines for human subject participation as outlined in the European GDPR and received formal approval from the Free University of Bozen‐Bolzano Research Ethics Committee (OWSM_Cod_2022_14). Given the sensitivity of the topics covered, including homophobic behaviors and bias‐based cyberbullying, measures were implemented to ensure participant well‐being. Participation was entirely voluntary, and participants were informed that they could skip any question they did not feel comfortable answering to minimize potential distress. Additionally, at the conclusion of the questionnaire, a list of psychological support services and LGBTQ+ organizations was provided, including helplines and online resources, ensuring that participants had access to relevant assistance if needed. Data collection was conducted using Qualtrics survey software (2020).

### 2.2 Measures

#### 2.2.1 Homophobic Cyberbullying

To investigate cyberbullying perpetration related to an individual's sexual orientation, we employed an adapted version of the European Cyberbullying Intervention Questionnaire (ECIPQ; Brighi et al. 2012). Our questionnaire consisted of a series of eight items that captured various aspects of cyberbullying perpetration based on sexual orientation. Participants were asked to indicate the frequency with which they engaged in these behaviors on a 5‐point Likert scale (from 0 = *Never* to 4 = *More than once per week*; Ω = 0.94). For detailed information on the items and the validity of the scale, please refer to the Supporting Materials.

#### 2.2.2 Social Dominance Orientation

SDO was measured using a scale comprised of eight items. This scale, originally developed by Pratto et al. (1994) and validated in Italian by Aiello et al. (2019), assesses the degree to which individuals endorse hierarchy‐enhancing legitimizing myths that favor dominant over subordinate groups. Participants expressed their agreement on a 5‐point Likert scale (0 = *Completely Disagree* to 4 = *Completely Agree*). Examples of items include, “Some groups of people are simply inferior to other groups,” and “It is a good thing that certain groups are at the top of our society and others at the bottom.” The scale demonstrated internal consistency (Ω = 0.89).

#### 2.2.3 Homophobic Social Norms

To measure the role of social norms in homophobic cyberbullying, we designed a 6‐item scale that investigates the role of negative peer attitudes toward the LGBTQ+ community. To ensure the validity of this scale, we followed the guidelines outlined in the Resources for Measuring Social Norms (Learning Collaborative to Advance Normative Change 2019). Participants responded on a 5‐point Likert scale (0 = *Completely Disagree* to 4 = *Completely Agree*). Example items were, “Most of my friends think that same‐sex marriage is not a right thing” and “Most of my friends believe that commenting on a photo by calling another person effeminate/masculine is acceptable.” The scale showed reliability (Ω = 0.84).

#### 2.2.4 Socio‐Emotional Competencies

In this study, we used the Socio‐Emotional Competencies Questionnaire (SEC‐Q; Zych et al. 2018), comprising 16 items rated on a Likert scale from 0 (*Strongly Disagree*) to 4 (*Strongly Agree*). The instrument evaluates four dimensions of SEC: self‐awareness (Ω = 0.72), self‐management (Ω = 0.73), prosociality (Ω = 0.71), and responsible decision‐making (Ω = 0.76). Sample items include “I understand how my emotions impact my actions” and “I generally listen actively.” The items were translated into Italian and subsequently back‐translated into Spanish to ensure accuracy. Consistent with the original scale development, a single composite score representing the mean of all subscales was calculated and used in this study.

### 2.3 Missing Data

Given the relatively high percentage of missing data (17.4%) resulting from online sampling, we conducted Little's (1988) test to assess the missing data mechanism. The test indicated that the missing data were missing completely at random (*p* = 0.832), supporting the appropriateness of multiple imputation. To address the missing data, we employed multiple imputation with 15 imputed data sets, following recommendations for handling missing data in social science research (McKnight et al. 2007). To ensure the validity of the imputed data, we conducted diagnostic checks, including comparing observed and imputed distributions to verify that imputation did not introduce systematic bias. Additionally, we examined convergence diagnostics to confirm that the iterative imputation process reached stability across data sets (Abayomi et al. 2008). The imputed data sets were then pooled for final analyses, ensuring that standard errors accounted for variability across imputations.

### 2.4 Analysis Plan

Due to the positive skewness of the homophobic cyberbullying variable (7.16), a log transformation was applied to improve normality and meet the assumptions of our statistical analyses (West 2022). This transformation helped reduce the impact of extreme values and ensured a more symmetrical distribution, minimizing potential bias in parameter estimation. To verify that the transformation did not distort the findings, we examined changes in skewness and kurtosis before and after the transformation and visually inspected Q–Q plots and histograms. The results confirmed that the transformed variable better approximated a normal distribution while maintaining the interpretability of the findings. In our preliminary analysis, we also estimated the prevalence of homophobic cyberbullying by categorizing individuals who reported engaging in at least one behavior at a frequency of “once a month” or more. This categorical classification—used for descriptive purposes only—was based on the adapted criteria from the ECIPQ scale and is detailed in the Supporting Materials.

Before conducting our focal analysis, we tested for differences in mean scores between the school‐based and online samples across all study variables using independent samples *t*‐tests. Results indicated statistically significant differences for all variables. However, given the consistent directionality of the effects and the theoretical rationale for examining homophobic cyberbullying across diverse adolescent groups as well as the generally small to medium effect sizes, we chose to pool the data to ensure adequate power and generalizability. Descriptive statistics by sample are reported in the Supporting Materials. For our focal analysis, a MANOVA was conducted to evaluate differences in the mean levels of all study variables across gender identity and sexual orientation groups. To assess the relationships between homophobic cyberbullying, SDO, HSN, and SEC, Pearson's bivariate correlations were performed.

Next, a series of multiple linear regression (MLR) models were fitted, given the continuous nature of the outcome variable. The initial model included only covariates, controlling for gender identity and sexual orientation. In the second model, key predictors were added. Finally, the third model tested two‐way interactions between the predictors, followed by a three‐way interaction. This final model was designed to examine whether SEC moderated the effect of HSN on the relationship between SDO and homophobic cyberbullying, allowing us to investigate how high and low levels of SEC influence this dynamic. In the main analyses (e.g., regression models), the homophobic cyberbullying scale was used as a continuous variable, calculated as the mean score across all seven items. All analyses were conducted using RStudio version 4.2.3.

## 3 Results

### 3.1 Descriptive Statistics and Preliminary Analysis

Means and standard deviation were generated and a MANOVA identified significant differences across gender identity and sexual orientation groups, (*F* (5, 1212) = 14.98, *p* < 0.001, *λ* = 0.79). Significant differences were found for SDO (*F* (5, 1872) = 16.94, *p* < 0.001, *η* ^2^ = 0.12), HSN (*F* (5, 757) = 18.94, *p* < 0.001, *η* ^2^ = 0.08), SEC (*F* (5, 2413) = 27.57, *p* < 0.001, *η* ^2^ = 0.05), and homophobic cyberbullying (*F* (5, 1638) = 9.17, *p* < 0.001, *η* ^2^ = 0.03). As predicted by H1, heterosexual boys reported the highest levels of homophobic cyberbullying (*M* = 0.16, SD = 0.42), supporting the expectation that gender and sexual orientation would influence involvement in homophobic cyberbullying. In addition, individuals identifying with a gender identity other than boys or girls reported the lowest levels of SEC (*M* = 2.17, SD = 0.58). See Table 1 for complete variable means and standard deviations. We conducted a correlation analysis to explore potential bivariate associations between the study variables. The results revealed a significant positive correlation between homophobic cyberbullying and SDO (*r* = 0.21, *p* < 0.001), as well as with HSN (*r* = 0.25, *p* < 0.001). Conversely, SEC were negatively correlated with homophobic cyberbullying (*r* = −0.06, *p* < 0.05). See Table 2 for the complete correlation matrix.

**Table 1:** Descriptive statistics of social dominance orientation, homophobic social norms, socio‐emotional competencies, and homophobic cyberbullying by gender identity and sexual orientation.

	Heterosexuals			Sexual minorities			*F*	*η* ^2^
	Boys	Girls	Other	Boys	Girls	Other		
	*M* (SD)	*M* (SD)	*M* (SD)	*M* (SD)	*M* (SD)	*M* (SD)		
Social dominance orientation	0.90 (0.86)	0.64 (0.66)	0.58 (0.59)	0.34 (0.45)	0.32 (0.46)	0.29 (0.44)	49.65***	0.12
Homophobic social norms	1.43 (0.86)	1.11 (0.77)	1.67 (0.60)	0.91 (0.82)	0.79 (0.75)	0.70 (0.74)	31.26***	0.08
Socio‐emotional competencies	2.91 (0.50)	2.82 (0.52)	2.17 (0.58)	2.64 (0.66)	2.65 (0.63)	2.45 (0.65)	27.57***	0.05
Homophobic cyberbullying	0.16 (0.47)	0.05 (0.26)	0.02 (0.01)	0.04 (0.12)	0.03 (0.19)	0.03 (0.07)	9.17***	0.03

*Note: F* represents the test statistic from the one‐way ANOVA. All variables ranged from 0 to 4.

Abbreviations: *M* = mean; SD = standard deviation.

*** *p* < 0.001.

**Table 2:** Spearman's correlation matrix.

Variables	1	2	3	4
1. Age				
2. Social dominance orientation	−0.13***	—		
3. Homophobic social norms	−0.13***	0.39***	—	
4. Socio‐emotional competencies	0.05**	−0.02	−0.05*	—
5. Homophobic cyberbullying	−0.04	0.35***	0.35***	−0.07*

*Note:* Significance levels:

*p < 0.05; **p < 0.01; ***p < 0.001.

### 3.2 Multiple Linear Regression Models

A series of MLR models, including interaction effects, were conducted to test our hypothesis. To account for the significant differences found in the preliminary analysis, gender identity and sexual orientation were included as control variables, along with age. Complete results from MLRs are displayed in Table 3.

**Table 3:** Multiple linear regression models of variables associated with homophobic cyberbullying.

	Model 1			Model 2			Model 3		
	*B*	SE	*p*	*B*	SE	*p*	*B*	SE	*p*
Intercept	0.05	0.01	< 0.001	0.03	0.03	0.376	0.38	0.16	0.022
Age	−0.01	0.01	0.552	−0.01	0.01	0.478	−0.01	0.01	0.505
Gender Identity^a^									
Gender (Boy)	0.04	0.01	< 0.001	–0.01	0.01	0.581	−0.01	0.01	0.537
Gender (Girl)	0.01	0.01	0.307	0.03	0.01	< 0.001	0.03	0.01	0.002
Sexual orientation^b^	–0.03	0.01	< 0.001	−0.03	0.01	< 0.001	−0.04	0.01	< 0.001
Social dominance orientation				0.26	0.01	< 0.001	0.25	0.10	0.011
Homophobic social norms				0.09	0.01	< 0.001	0.09	0.04	0.036
Socio‐emotional competencies				−0.21	0.01	0.038	−0.19	0.07	0.007
Two way interactions									
Social dominance orientation × Homophobic social norms							0.06	0.03	0.034
Social dominance orientation × Socio‐emotional competencies							0.15	0.04	< 0.001
Social norms × Socio‐emotional competencies							0.05	0.02	0.013
Three way interaction									
Social dominance orientation × Homophobic social norms × Socio‐emotional competencies							−0.03	0.01	0.001
*R* ^2^	2.5			11.3			15.4		

Abbreviations: *B* = unstandardized regression coefficients; SE = standard errors.

^a^ For gender identity, those who identified with other then boy or girl served as reference group.

^b^ For sexual orientation, heterosexuals served as reference group.

In the covariate‐only model (Model 1), boys reported higher levels of homophobic cyberbullying compared to girls, and heterosexual youth reported higher rates of homophobic cyberbullying. As expected by H2 and H3, the model including risk and protective factors (Model 2) showed that SDO was positively associated with homophobic cyberbullying (*B* = 0.26, *p* = 0.011), while HSN was also positively associated (*B* = 0.09, *p* = 0.036). In contrast, SEC were negatively associated with homophobic cyberbullying (*B* = −0.21, *p* = 0.007).

#### 3.2.1 Moderation Analysis

**Figure 1:**
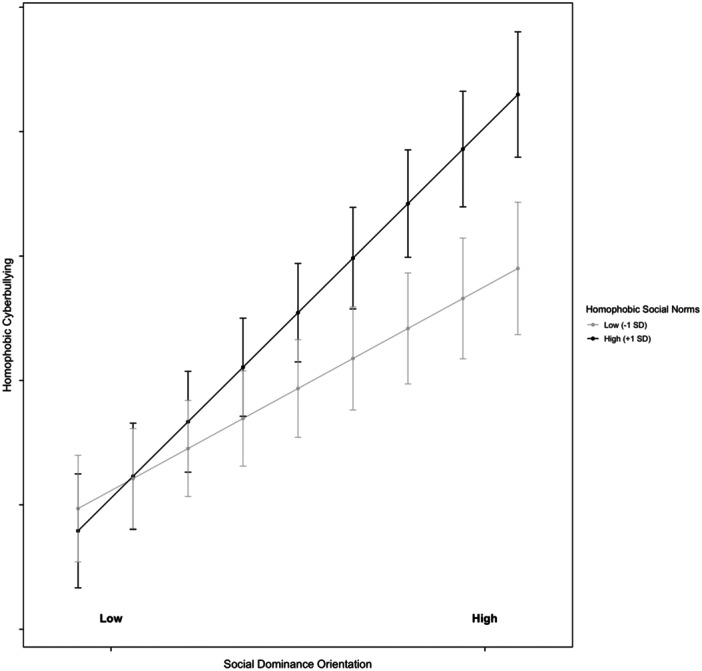
Moderating effect of homophobic social norms on the relationship between social dominance orientation and homophobic cyberbullying. Low homophobic social norms: 1 SD below the mean; high homophobic social norms: 1 SD above the mean.

**Figure 2:**
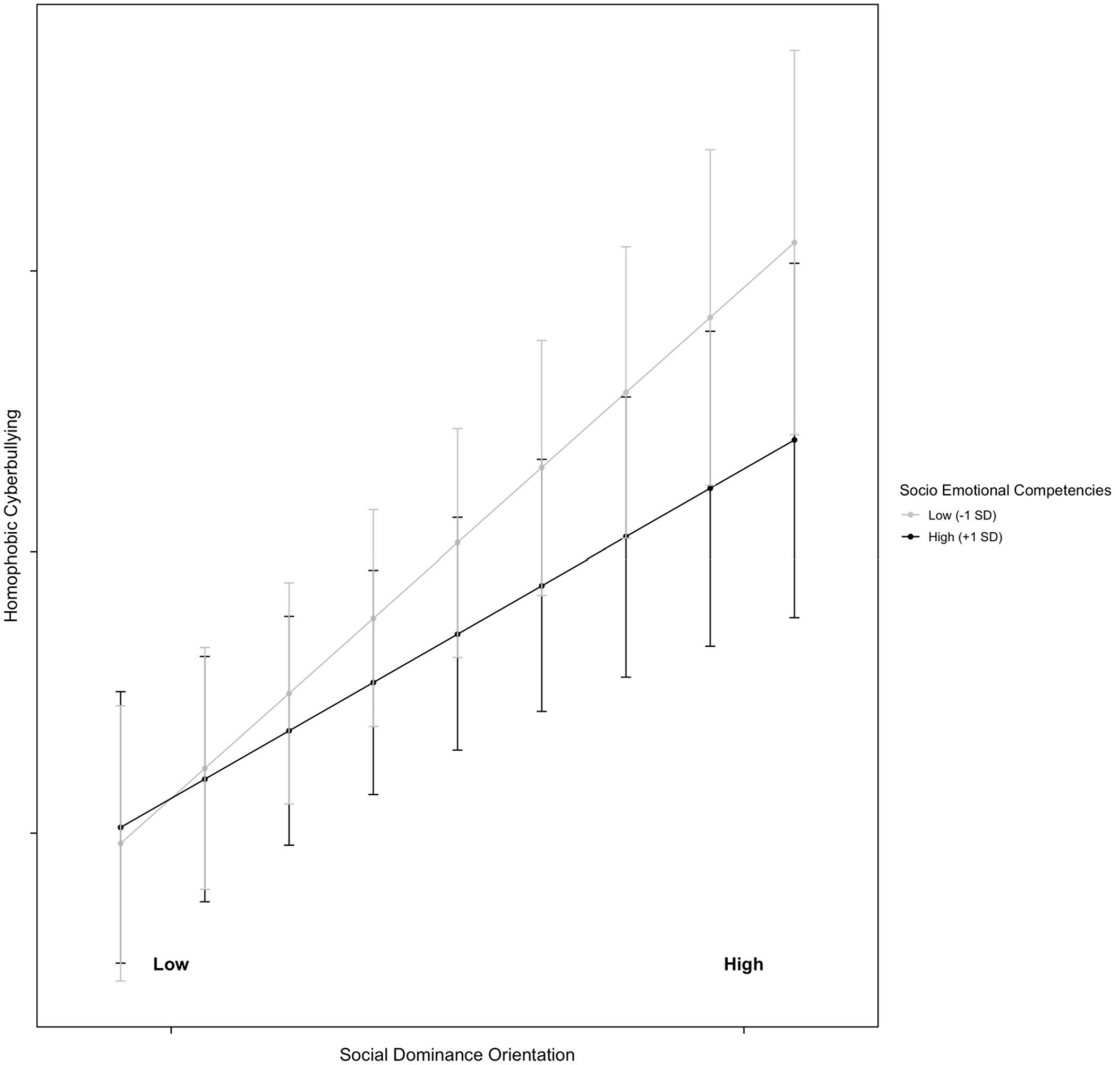
Moderating effect of socio‐emotional competencies on the relationship between social dominance orientation and homophobic cyberbullying. Low socio‐emotional competencies: 1 SD below the mean; high socio‐emotional competencies: 1 SD above the mean.

**Figure 3:**
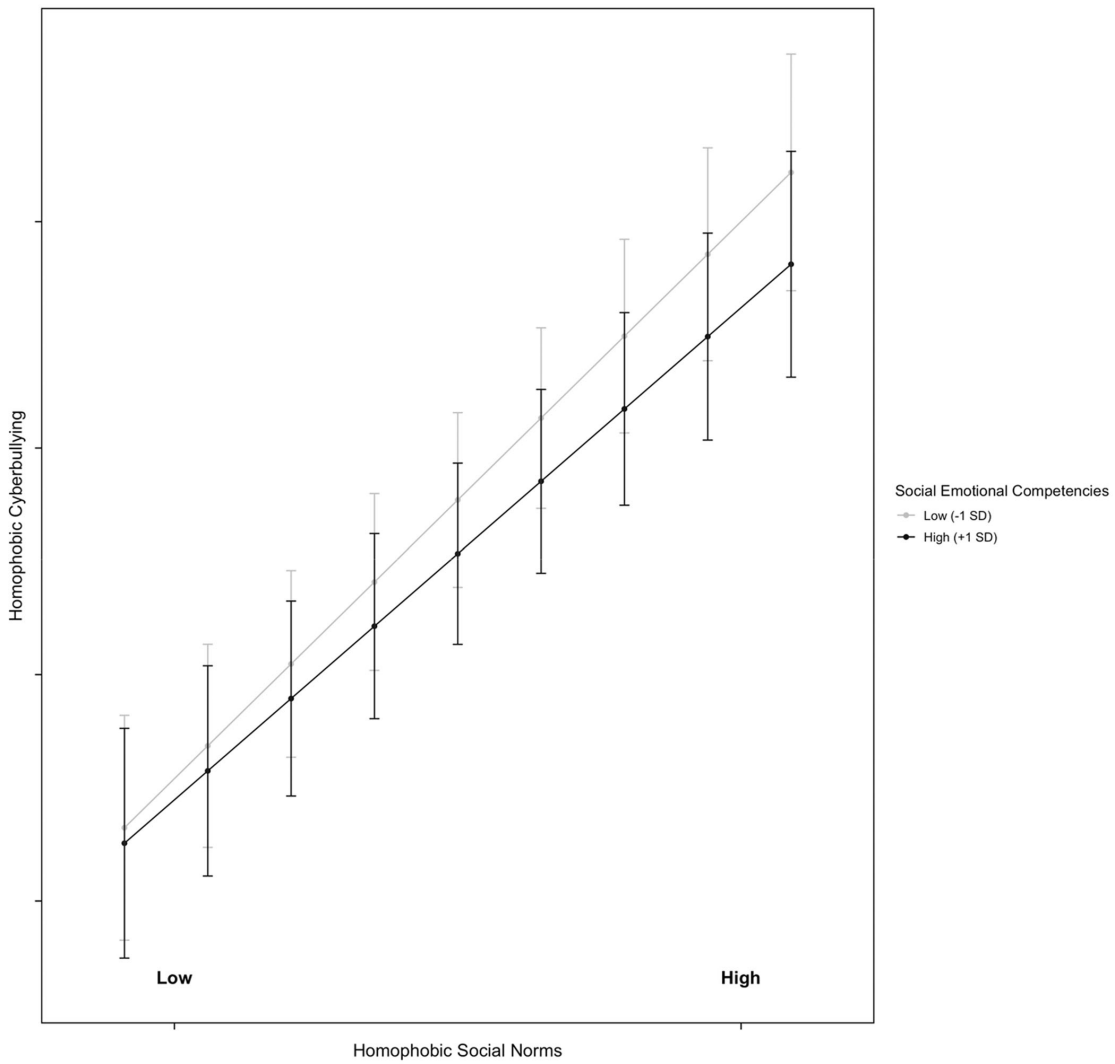
Moderating effect of socio‐emotional competencies on the relationship between homophobic social norms and homophobic cyberbullying. Low socio‐emotional competencies: 1 SD below the mean; high socio‐emotional competencies: 1 SD above the mean.

The subsequent model examined whether the association between norms and homophobic cyberbullying varied depending on adolescents' SEC (Model 3). Specifically, we tested a series of models that included two‐way and three‐way interactions between SEC, HSN, and SDO. First, we analyzed the two‐way interaction effects: (1) SDO × HSN (*B* = 0.06, SE = 0.03, *p* = 0.034), (2) SDO × SEC (*B* = 0.15, SE = 0.04, *p* < 0.001), and (3) HSN × SEC (*B* = 0.05, SE = 0.02, *p* = 0.013). As predicted by H4, when HSN was one standard deviation above the mean, the effect of SDO on homophobic cyberbullying was significant (*B* = 0.05, SE = 0.01, *p* < 0.05), whereas when HSN was one standard deviation below the mean, the effect was nonsignificant (*B* = −0.01, SE = 0.01, *p* = 0.472). The results for the first interaction effect are illustrated in Figure 1. For the second interaction effect, adolescents with higher levels of SEC exhibited a weaker effect of SDO on homophobic cyberbullying (*B* = 0.02, SE = 0.01, *p* < 0.05), compared to those with lower SEC, where the effect was stronger (*B* = 0.04, SE = 0.01, *p* < 0.01). The results for the second interaction effect are illustrated in Figure 2. Similarly, for the third interaction effect, higher levels of SEC were associated with a reduced influence of HSN on homophobic cyberbullying (*B* = 0.03, SE = 0.01, *p* < 0.01), whereas lower SEC were linked to a stronger effect (*B* = 0.04, SE = 0.01, *p* < 0.01). The results for third interaction effect are illustrated in Figure 3.

**Figure 4:**
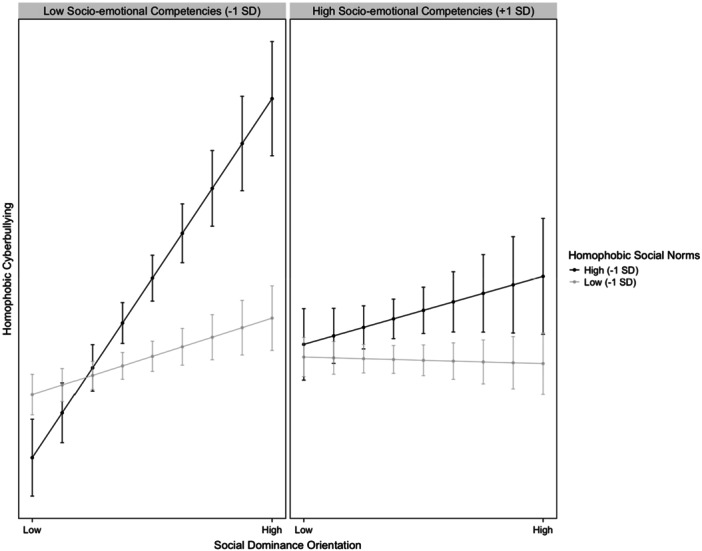
Three‐way interaction effect of socio‐emotional competencies, homophobic social norms, and social dominance orientation on homophobic cyberbullying. Low homophobic social norms: 1 SD below the mean; high homophobic social norms: 1 SD above the mean. Low socio‐emotional competencies: 1 SD below the mean; high socio‐emotional competencies: 1 SD above the mean.

The next step involved testing whether the moderating effect of HSN on the association between SDO and homophobic cyberbullying would vary based on adolescents' levels of SEC. Given that all the two‐way interactions were significant, a three‐way interaction was examined between SEC, HSN, and SDO (see Model 3 in Table 3). As illustrated in Figure 4, for adolescents with SEC one standard deviation below the mean, the effect of SDO on homophobic cyberbullying was significantly higher among those with higher levels of HSN (*B* = 0.06, SE = 0.01, *p* = 0.004), while the slope for those with lower HSN was not significant (*B* = −0.01, SE = 0.01, *p* = 0.342). However, for adolescents with SEC one standard deviation above the mean, the effect of SDO on homophobic cyberbullying remained significant but lower for those with higher HSN (*B* = 0.03, SE = 0.01, *p* < 0.01). In contrast, for those with lower HSN, the slope was again nonsignificant (*B* = −0.01, SE = 0.01, *p* = 0.632).

To summarize, the three‐way interaction model shows that adolescents with lower SEC and higher HSN supporting cyberbullying are more likely to engage in homophobic cyberbullying when influenced by high levels of SDO. Conversely, those with higher SEC are less affected by HSN and SDO, indicating that socio‐emotional skills can buffer against the influence of harmful norms and individual biases on cyberbullying behaviors.

## 4 Discussion

The present study aimed to examine the relationships between HSN, SDO, and homophobic cyberbullying among adolescents, while exploring the moderating role of SEC. Research on the risk and protective factors of bias‐based cyberbullying is crucial, as social norms and power dynamics in digital environments can normalize homophobic behaviors. Our findings indicate that adolescents with higher levels of SEC were less influenced by these negative norms and individual biases, suggesting that SEC can serve as a protective factor against homophobic cyberbullying perpetration.

Our initial hypotheses center on examining the prevalence of homophobic cyberbullying among different groups. Descriptive analyses showed that heterosexual boys were significantly more likely to be perpetrators. While the literature on generic cyberbullying yields mixed results regarding gender roles, our findings align with prior research highlighting heterosexual boys as the primary bias‐based aggressors, particularly as it relates to homophobic cyberbullying (Poteat et al. 2013, 2015). Masculine gender identity has been shown to predict involvement in cyberbullying, including general, religion‐based, and race‐based forms (Hinduja and Patchin 2022). Similarly, among Spanish youth, heterosexual boys reported the highest rates of bias‐based cyberaggression, more than second‐generation immigrants and sexual minorities (Ojeda et al. 2023; Zych and Llorent 2023).

The consistent association between aggressive behavior and normative masculine attitudes (Poteat et al. 2011; Wright and Wachs 2020) may be explained by the assumption that homophobia involves sexually prejudiced interactions reinforcing gendered and racialized inequalities (Pascoe 2013) within the framework of hegemonic masculinity (Kimmel and Kaufman 1993). Lipman‐Blumen (1976) and Kimmel and Kaufman (1993) theorized that homophobia is inherently tied to masculinity, with normative attitudes reinforcing a hegemonic concept of “manhood” that drives aggressive behavior.

Our second hypothesis focused on the role of contextual factors (HSN) that might influence homophobic cyberbullying. Contrary to previous findings (Poteat et al. 2013), the level and effect of negative peer attitudes toward the LGBTQ+ community were higher among heterosexual boys than heterosexual girls. Peer norms, particularly among heterosexual boys, may incorporate homophobic behavior as humor, increasing engagement in homophobic cyberbullying (Pascoe 2013; Poteat et al. 2015). As suggested by our findings, adolescents may internalize homophobic norms within their social circles, potentially increasing their likelihood of engaging in homophobic cyberbullying. Building on social identity theory (Tajfel and Turner 1979), biased individuals may resist being misclassified into a group (e.g., sexual minorities) due to their strong negative attitudes, possibly influenced by interpersonal dynamics among peers (Poteat 2007). Reducing prejudice through addressing these norms is essential, as both cyberbullying and prejudice contribute to the mental health and academic challenges faced by sexual minority youth (Matsick et al. 2020; Stacey and Wislar 2023).

In line with social dominance theory (Pratto et al. 1994; Sidanius and Pratto 1999), our third hypothesis proposed that SDO would be positively associated with homophobic cyberbullying. The results supported this hypothesis, confirming that individuals with higher SDO are indeed more likely to engage in discriminatory behaviors, including homophobic cyberbullying. This finding aligns previous research linking SDO with various forms of biased aggression, such as cyberbullying and hate speech (O'Brien et al. 2013; Licciardello et al. 2014). These results highlight the broader role of social dominance in fostering online aggression, particularly among those who endorse hierarchical social structures. Further, previous studies have shown that SDO is strongly correlated with increased engagement in cyberbullying especially when such behaviors are reinforced by peer approval (McInroy 2020; Wang 2022). The role of social norms in shaping this behavior reinforces the idea that homophobic cyberbullying tends to escalate in environments where negative attitudes toward LGBTQ+ individuals are pervasive. This connection between SDO, HSN, and homophobic cyberbullying is critical for understanding how power dynamics and peer influences shape adolescents' aggressive behavior in digital spaces.

In accordance with our fourth hypothesis, the study found that SEC served as a protective factor, buffering the effect of HSN on SDO in relation to homophobic cyberbullying. This finding aligns with previous research that has consistently shown a link between low SEC and increased aggression (Arató et al. 2020; Zych et al. 2018), providing further evidence that adolescents with underdeveloped SEC are more vulnerable to engaging in aggressive behaviors. What is particularly novel about our findings is the demonstration that SEC not only influences individual aggression but can actively moderate the interaction between HSN and SDO, reducing the likelihood of homophobic cyberbullying in environments where prejudiced norms are dominant. While previous research has focused on the direct effects of SEC (Estévez et al. 2019; Zych et al. 2018, 2019), our study highlights its moderating role in bias‐based aggression. Moreover, this interaction was more pronounced in certain social contexts, suggesting that the protective effect of SEC may be context‐dependent, influenced by peer group dynamics and the reinforcement of homophobic norms (Romer et al. 2011).

Given the role of SEC in buffering the effects of HSN and SDO on homophobic cyberbullying, intervention programs should incorporate targeted socio‐emotional learning strategies to reduce bias‐based aggression. Research suggests that programs fostering empathy, emotional regulation, and conflict resolution skills can effectively reduce bias‐driven behaviors and promote inclusivity (de Mooij et al. 2024). For instance, the HateLess intervention has been shown to increase empathy and counter‐speech efficacy in addressing online hate speech (Wachs et al. 2023). Similarly, social‐emotional learning programs implemented in school curricula have demonstrated positive effects on students' social interactions, academic outcomes, and reduction of aggressive behaviors (Hangartner et al. 2021; de Mooij et al. 2024). Additionally, perspective‐taking exercises and empathy training have been linked to reductions in prejudice and stereotyping, reinforcing their value in anti‐cyberbullying initiatives (Rodríguez Chatruc and Rozo 2024). Integrating these approaches into school policies and digital safety education could help counteract the normalization of homophobic cyberbullying and foster a more inclusive peer culture both online and offline.

Lastly, while our findings showed statistically significant relationships HSN, SDO, SEC, and homophobic cyberbullying, the effect sizes were generally relatively small. This pattern is not uncommon in research on bias‐based cyberbullying, where behaviors are shaped by a combination of individual traits, peer influences, and broader societal norms. Even small effects, however, can accumulate over time and have meaningful consequences at the population level (Matthay et al. 2021), particularly in school settings where peer norms and social hierarchies strongly influence adolescent behavior. The finding that SEC moderates the effects of SDO and HSN on homophobic cyberbullying, albeit with small effect sizes, suggests that enhancing SEC at a broader scale could still contribute to reducing bias‐based aggression in educational and online environments. Furthermore, small effect sizes in social science research do not imply that findings lack practical relevance. In studies on prejudice, aggression, and cyberbullying, even modest changes in social norms, interventions, or educational programs can lead to meaningful shifts in behavior over time. These results emphasize the importance of comprehensive interventions—approaches that target both individual competencies (e.g., SEC) and social structures (e.g., school policies, peer dynamics, and digital platform regulations)—to effectively mitigate homophobic cyberbullying (Abreu and Kenny 2017; Amadori et al. 2023).

### 4.1 Limitations and Future Directions

This study benefits from a large sample of sexual minorities, allowing for clearer insights into the differences between adolescents based on both gender and sexual orientation. This is one of the first studies to achieve this level of detail. Through the use of a multimethod sampling (i.e., collecting data from both schools and online platforms) this study may have reached sexual minority youth who may be more likely to miss school due to feeling unsafe or marginalized (Russell et al. 2014), ensuring a more representative and less bias sample. However, the study also has noteworthy limitations. First, its cross‐sectional nature prevented us from inferring causality between the observed variables and homophobic cyberbullying. Future research should explore longitudinal associations between social dominance and peer influence on homophobic behavior online. Second, this study used observed variable analysis instead of latent variable modeling. While this choice allows for straightforward interpretation, it does not account for potential measurement error in constructs like SEC, SDO, and HSNs. Future research could consider using latent variable approaches, such as structural equation modeling, to improve the accuracy of these measurements and explore the relationships between psychological factors in more depth. This approach would also make it possible to test more complex models, including indirect and mediated effects, offering a broader perspective on the mechanisms behind homophobic cyberbullying. Third, the age range of participants (14–19 years) may limit the generalizability of findings to younger populations. Since homophobic bullying often begins in preadolescence and is particularly prevalent in middle schools (Espelage et al. 2019), future research should examine these associations among younger age groups to identify early risk and protective factors. Expanding the age range would provide a more complete picture of how social dominance, peer norms, and SECs influence homophobic cyberbullying across different developmental stages. Fourth, the specificity of certain items in the homophobic cyberbullying scale may have influenced response patterns. In particular, Item 6 (“insulted/offended someone on online gaming platforms due to their sexual orientation”) referred to a specific digital space, whereas other items assessed behaviors more generally across online platforms. This difference may have led to underreporting among participants who engaged in similar behaviors in nongaming contexts (e.g., social media, messaging apps). Future research should consider generalizing this item to better capture homophobic cyberbullying across multiple digital environments, ensuring greater comparability across all measures. Further, while our study focused exclusively on online experiences, we acknowledge that bias‐based aggression is not confined to digital spaces. Research suggests that SDO and SEC are relevant to both online and offline contexts. However, due to parsimony and resource constraints, we did not control for in‐person biases or general cyberbullying perpetration. Future research should examine whether the relationships found in this study persist when accounting for these factors. Finally, while significant differences emerged between the school‐based and online subsamples across study variables, we chose to pool the data to reflect the diversity of Italian adolescents and maximize statistical power. Future studies might explore whether these associations differ by context more explicitly.

## 5 Conclusions

This study highlights the value of using a socioecological stigma framework to understand bias‐based cyberaggression among adolescents. Our findings suggest that online homophobia stems from a complex interplay of individual (e.g., inclination toward social dominance) and contextual factors (e.g., peer group social norms). Our findings can inform the development of inclusive prevention programs for both bullying and cyberbullying. Existing research strongly supports the effectiveness of programs that incorporate a Social and Emotional Learning (SEL) perspective. Research has shown that such programs can significantly reduce bullying and cyberbullying by equipping young people with the socio‐emotional skills needed to address such issues (Amadori et al. 2023). By incorporating SEL school prevention programs can be more effective in combating cyberbullying and creating inclusive online environments for all adolescents. Further research is needed to explore the most impactful methods for integrating these findings into educational and community‐based programs.

## Ethics Statement

The project associated with this study received approval from the Research Ethics Committee of the Free University of Bozen‐Bolzano (Protocol Number: OWSM_Cod_2022_14).

## Conflicts of Interest

The authors declare no conflicts of interest.

## Supporting information


**Table S1**. Descriptive statistics for homophobic cyberbullying.
**Table S2**. Descriptive statistics of study variables by sampling procedure.

## Data Availability

The authors are unable to share the data as they do not have permission to do so.
