# Supplementary material for: Risks and Protective Factors Associated to Homophobic Cyberbullying Among Youth
Source: Aggress Behav. 2025 May 21;51(3):e70034. doi: 10.1002/ab.70034 (PMC12095989; doi:10.1002/ab.70034)
Supplement: Supplementary file 1 — Table S1. Descriptive statistics for homophobic cyberbullying. Table S2. Descriptive statistics of study variables by sampling procedure. [file AB-51-e70034-s001.docx]

**Supplementary materials**

**Preliminary analysis**

In our preliminary analysis, we tested a measurement model via CFA to assess the construct validity of the adapted ECIPQ scale. Model fit was evaluated using the comparative fit index (CFI; > 0.90), Tucker–Lewis Index (TLI; > 0.90), root mean square error of approximation (RMSEA; < 0.06), and the standardized root mean square residual (SRMR; < .08; Hu & Bentler, 1999). For parameter estimation, we used weighted least squares with mean and variance adjustments (WLSMV), a robust variant of diagonally weighted least squares (DWLS), which is suitable for ordinal data (Li, 2016). The results of the CFA indicated strong fit indices, confirming the validity of the scale: CFI = 0.996, TLI = 0.994, RMSEA = 0.051, and SRMR = 0.055. These results suggest that the adapted scale reliably assesses the construct of homophobic cyberbullying in the sample, supporting its use in further analyses.

A descriptive analysis of homophobic cyberbullying revealed that the most common forms involved offenses related to someone's sexual orientation. For simplicity and clarity, Likert scale points 1-2 (once or twice – once a month) and 3-4 (once a week, more than once a week) were combined (Table S1). In the past year, 5.68% of participants reported involvement in homophobic cyberbullying incidents, such as posting someone else's sexual orientation online, while 5.2% mentioned gossiping about someone’s sexual orientation. More frequent aggression was observed in 1.38% of participants, who insulted or offended others on online gaming platforms due to their sexual orientation more than once a week. In order to provide an estimate of prevalence, we relied on a categorical classification of perpetrators, adapted from the original ECIPQ protocol. In the original scale, perpetration was defined as scoring ≥ 2 ("once a month") on any perpetration item, while not simultaneously scoring ≥ 2 on any victimization item. As our study did not include victimization data, we adopted a conservative approximation: participants were identified as homophobic cyberbullying perpetrators if they reported a frequency of once a month or more (score ≥ 2) on at least one of the homophobic perpetration items. In total, 144 participants (3.7%) were identified as homophobic cyberaggressors.

To assess potential differences between the online and school-based samples, we conducted independent t-tests on all study variables (see Table S2). Results indicated statistically significant differences across groups; however, effect sizes were generally small to moderate (Cohen’s d ranging from 0.16 to 0.63). These findings support the decision to combine both samples in the main analyses while acknowledging group differences.

**Table S1**

*Descriptive Statistics for Homophobic Cyberbullying*

| Item | Never | Once a Month | More Than Once a Week | Factor Loading |
| --- | --- | --- | --- | --- |
|  | *n* (%) | *n* (%) | *n* (%) |  |
| 1. Said unpleasant things/offended someone online due to their sexual orientation. | 3315 (93.22%) | 202 (5.68%) | 39 (1.1%) | 0.71 |
| 1. Spoke negatively about someone online due to their sexual orientation. | 3314 (93.51%) | 186 (5.25%) | 44 (1.24%) | 0.75 |
| 2. Created a fake account to tease someone because of their sexual orientation. | 3477 (98.19%) | 43 (1.21%) | 21 (0.59%) | 0.79 |
| 3. Published information online about someone's sexual orientation. | 3378 (95.26%) | 142 (4%) | 26 (0.73%) | 0.73 |
| 4. Posted embarrassing videos/photos online because someone was perceived as LGBTQ+. | 3451 (97.43%) | 70 (1.98%) | 21 (0.59%) | 0.75 |
| 5. Excluded/ignored someone on social networks/online gaming because of their sexual orientation. | 3402 (96.43%) | 95 (2.69%) | 31 (0.88%) | 0.82 |
| 6. Insulted/offended someone on online gaming platforms due to their sexual orientation. | 2177 (96.84%) | 40 (1.78%) | 31 (1.38%) | 0.80 |

*Note.* The table provides the frequency and percentage of respondents who reported engaging in each behavior once a month or more than once a week. Percentages are calculated based on the total number of responses for each frequency category. The question posed for each item was: “During the past 12 months, how many times have you…”

**Table S*2***

*Descriptive Statistics of Study Variables by Sampling Procedure*

|  | Online | School | *t*(3807) | Cohen's *d* |
| --- | --- | --- | --- | --- |
|  | *M (SD)* | *M (SD)* |  |  |
| Social dominance orientation | 1.32 (0.48) | 1.73 (0.76) | 16.32*** | 0.63 |
| Homophobic social norms | 1.80 (0.79) | 2.02 (0.83) | 11.88*** | 0.49 |
| Socio emotional competencies | 3.55 (0.66) | 3.78 (0.56) | 10.77*** | 0.37 |
| Homophobic Cyberbullying | 0.03 (0.12) | 0.06 (0.21) | 4.41*** | 0.16 |

*Note*. *** *p* < .001
